# Circulating microRNAs as Diagnostic Markers in Primary Aldosteronism

**DOI:** 10.3390/cancers13215312

**Published:** 2021-10-22

**Authors:** Scott M. MacKenzie, Hannah Saunders, Josie C. van Kralingen, Stacy Robertson, Alexandra Riddell, Maria-Christina Zennaro, Eleanor Davies

**Affiliations:** 1 British Heart Foundation Glasgow Cardiovascular Research Centre (BHF GCRC), Institute of Cardiovascular & Medical Sciences (ICAMS), University of Glasgow, Glasgow G12 8TA, UK; hannahsaunders1@sky.com (H.S.); Josie.VanKralingen@glasgow.ac.uk (J.C.v.K.); Stacy.Robertson@glasgow.ac.uk (S.R.); Alexandra.Riddell@glasgow.ac.uk (A.R.); eleanor.davies@glasgow.ac.uk (E.D.); 2 Paris-Cardiovascular Research Center (PARCC), Institut National de la Santé et de la Recherche Médicale (INSERM), Université de Paris, 75015 Paris, France; maria-christina.zennaro@inserm.fr; 3 Assistance Publique-Hôpitaux de Paris, Hôpital Européen Georges Pompidou, Service de Génétique, 75015 Paris, France

**Keywords:** primary aldosteronism, microRNA, aldosterone, circulating, biomarker, adrenocortical

## Abstract

**Simple Summary:**

Many patients remain at increased risk of primary aldosteronism (PA) and its consequences due to the difficulty of accurate diagnosis. MicroRNAs circulating in the bloodstream are emerging as biomarkers for disease, particularly specific forms of cancer. In this review article, we argue that they may also have a role in the diagnosis of PA, if observed changes in the microRNA profile of PA tissue are reflected in circulating microRNAs, which can be sampled and analysed readily in a clinical setting. However, for various practical reasons, studies of potential diagnostic circulating microRNAs have often proved difficult to reproduce consistently. We describe these problems and how they might be overcome using, as an example, our design of the circulating microRNA arm of the ongoing ENS@T-HT project, which is intended to confirm whether circulating microRNAs can serve as biomarkers for PA.

**Abstract:**

Primary aldosteronism (PA) is a common and highly treatable condition, usually resulting from adrenocortical tumorous growth or hyperplasia. PA is currently underdiagnosed owing to its complex and protracted diagnostic procedures. A simplified biomarker-based test would be highly valuable in reducing cardiovascular morbidity and mortality. Circulating microRNAs are emerging as potential biomarkers for a number of conditions due to their stability and accessibility. PA is known to alter microRNA expression in adrenocortical tissue; if these changes or their effects are mirrored in the circulating miRNA profile, then this could be exploited by a diagnostic test. However, the reproducibility of studies to identify biomarker-circulating microRNAs has proved difficult for other conditions due to a series of technical challenges. Therefore, any studies seeking to definitively identify circulating microRNA biomarkers of PA must address this in their design. To this end, we are currently conducting the circulating microRNA arm of the ongoing ENS@T-HT study. In this review article, we present evidence to support the utility of circulating microRNAs as PA biomarkers, describe the practical challenges to this approach and, using ENS@T-HT as an example, discuss how these might be overcome.

## 1. Introduction

The autonomous production of aldosterone by the adrenal gland, as observed in cases of primary aldosteronism (PA), adds significantly to the overall burden of cardiovascular risk, owing to the raised blood pressure and damage to vascular and other tissues that result from elevated circulating levels of the hormone. The majority of cases can be attributed to tumorous growth or hyperplasia of the adrenal cortex: the development of an aldosterone-producing adenoma (APA) on one gland results in lateralised oversecretion of aldosterone, while bilateral adrenal hyperplasia (BAH) causes elevated hormone production from both. These two subtypes account for ~90% of cases, with the remainder consisting of familial forms of PA or the less common unilateral form of hyperplasia [1]. Significant progress has been made in recent years to identify key mutations driving the pathogenesis of PA. APA are commonly found to have somatic mutations to the *KCNJ5* and *CACNA1D* genes or, less frequently, other genes including *ATP1A1*, *ATP2B3* and *CTNNB1* [2]. In cases of hyperplasia, there is now also strong evidence that mutation to certain of these same genes—particularly *CACNA1D*—could drive overproduction of aldosterone from small, distinct groups of cells within the cortex, termed aldosterone-producing cell clusters (APCC) [3].

Despite being acknowledged as the most common form of secondary hypertension, there is growing recognition that the currently accepted prevalence level of PA (5–10% of hypertensive patients) is likely to be a significant underestimation, with authorities in the field suggesting a true figure some 3 to 5 times higher [4]. That so many PA patients currently evade diagnosis is largely attributable to two main factors: firstly, the failure to refer hypertensive patients for PA screening tests and, secondly, the overly stringent and/or technically demanding nature of those same screening tests—and subsequent confirmatory tests—which limits throughput while also returning high rates of false negative results [5]. With a growing body of evidence showing that many PA cases may not be accompanied by an elevated aldosterone-renin ratio (ARR), hypokalaemia or even high blood pressure [6,7], accurate diagnosis of PA appears ever more difficult. Ongoing adaptations to existing tests that recognise, for example, the importance of 24 hr aldosterone measurement over a single spot test, or the impact of ACTH as well as the renin angiotensin system on aldosterone secretion, may result in some improvements to the situation, but diagnosis by these means will still remain highly demanding in terms of time, labour and cost. However, improving diagnosis to identify even a fraction of the currently undiagnosed cases would reap huge benefits, given that PA is highly responsive to treatment and its major consequences can be reduced or cured through adrenalectomy or administration of mineralocorticoid receptor antagonists (MRAs). The development of a simple high-throughput blood test for PA, utilising the measurement of one or more specific biomarkers, is therefore a highly attractive goal, albeit one that may previously have seemed somewhat remote.

A good biomarker should show high specificity and sensitivity for the intended disease state, be accessible through its presence in peripheral tissue or fluids (e.g., blood, saliva, urine, etc.) and be easily detected and/or quantified by robust, rapid and affordable assays. In light of the promise that circulating miRNA (c-miRNA) has shown as a biomarker in the diagnosis of other conditions, and the current evidence that PA results in changes to the levels of particular microRNAs, both in tissue and in the bloodstream, in this article, we discuss the prospect of using these molecules as the basis of a future PA diagnostic test. Additionally, given the common technical problems that have frustrated attempts to discover diagnostic c-miRNAs for other conditions, we examine how these might be addressed and hopefully overcome in the case of PA, with particular reference to our involvement in the ENS@T-HT project, an ongoing study that includes, among its aims, the identification of diagnostic c-miRNAs for PA and other forms of endocrine hypertension.

## 2. MicroRNA

MiRNA is a class of small, non-coding RNA (ncRNA) approximately 22 nucleotides long. The significant role of these molecules in gene expression was first identified in *C. elegans* some thirty years ago [8]. They are now known to have a crucial role in the development and regulation of key biological systems across all animals (and plants), with conservation of many of these processes across species. The current miRbase release (www.mirbase.org, accessed on 30 August 2021, v.22) from March 2018 lists 1115 different annotated mature human miRNAs, although a recent study estimates the true total number to be as high as 2300 [9]. The majority of miRNAs are transcribed from intra- and intergenic regions of DNA as pri-miRNAs, undergoing processing by RNAses to form pre-miRNAs and, finally, the mature miRNA itself. They generally act as repressors of gene expression, interacting through base complementarity with the 3’UTR of specific target messenger RNAs (mRNAs) to promote their degradation and prevent their translation [10]. This process is mediated via the RNA-induced silencing complex (RISC), a ribonucleoprotein complex that incorporates the miRNA alongside the Argonaute 2 (AGO2) protein to enable interaction with the mRNA. While instances have been reported of miRNAs interacting with other regions of the mRNA, and even of their stimulating gene expression, this repressive mode of miRNA action via the mRNA 3’UTR appears by far the most common. The interaction between the seed region of a miRNA (nucleotides 2–8) and its target mRNA is key, and this aspect of miRNA action has been exploited to develop specific bioinformatic algorithms that predict mRNA/gene targets, often on the basis of the miRNA seed sequence [11]. These predictions are far from perfect and are no substitute for experimental validation of miRNA action but can serve as useful tools in guiding such validation studies [12]. Given the short length of miRNAs, and the even shorter length of the seed region, an individual miRNA within a cell has the potential to target many different mRNA species that each harbour a complementary sequence within their 3’UTRs. Therefore, by producing just a single miRNA, the cell has the ability to target the expression of numerous genes simultaneously, making them a potentially powerful pleiotropic mediator of biological processes. Furthermore, given their post-transcriptional mode of action, a particular miRNA expressed in two or more different tissues may target a completely different array of mRNAs in each, due to differences in their respective transcriptomes. To fulfil their key regulatory role, production of miRNAs themselves must be tightly controlled so as to restrict their presence to particular tissues and/or specific biological conditions. Abnormal expression of miRNA—perhaps through loss of genetic or epigenetic control of pri-miRNA transcription, or faults in miRNA processing—can result in disease. Such changes in tissue levels of specific miRNAs within a tissue have been associated with various forms of cancer [13]. While such findings provide valuable insight into the mechanism and possible treatment of such conditions, the ability to detect, distinguish and quantify precisely the different miRNA species also means that they represent a potentially valuable diagnostic tool. For diagnostic purposes, it is irrelevant whether such changes in miRNA levels are the cause or the consequence of that condition. As with so many approaches based on diagnostic biomarkers, a major drawback to analysing tissue miRNAs is often the difficulty of obtaining tissue samples from the patient for analysis. In the case of miRNAs, however, this difficulty may be avoided due to their presence in extracellular fluids.

Low levels of miRNA have been detected in various bodily fluids, including serum, plasma, urine and breast milk. In plasma, they are found mostly in the form of RISC/AGO2 complexes, although they also associate with extracellular vesicles (EVs) or high-density lipoproteins; these structures apparently protect the miRNAs from RNase degradation [14,15]. The majority of extracellular miRNAs are thought to derive from the passive leakage of dead or apoptotic cell contents into the extracellular space and, to a lesser extent, from the packaging of miRNAs into EVs such as exosomes, microvesicles and apoptotic bodies, which are then actively secreted from the cell, possibly as a form of intercellular communication. Regardless of their source, the discovery of such miRNAs quickly prompted speculation that they might have value as circulating diagnostic biomarkers for specific disease states.

This hypothesis gained significant credibility following work by Mitchell et al. in 2008 [16]. Demonstrating the remarkable stability of these nucleic acids in clinical plasma and serum samples, they proposed miRNAs to be a novel class of blood-based cancer biomarkers well suited to that role given the common dysregulation of miRNA expression in cancers and the tissue-specific nature of such miRNA changes. To support their case, they detected an elevation of hsa-miR-141-3p levels in the serum of prostate cancer patients relative to healthy controls. Over subsequent years, numerous studies have emerged attempting to correlate c-miRNA profiles with particular diseases and, while different forms of cancer (including lung, breast, prostate, liver and ovarian) have undoubtedly been the main focus of such work, the principles underlying this concept can be expanded to encompass any disease affecting tissue miRNA expression, including diabetes, rheumatoid arthritis, Parkinson’s disease, Alzheimer’s disease and various forms of cardiovascular disease such as heart failure, coronary artery disease and stroke [17].

## 3. Adrenal miRNA in Adrenal Steroidogenesis and PA

The case for c-miRNAs as viable biomarkers for PA is supported by the regulatory role miRNAs play in the processes disrupted by PA—i.e., adrenal production of aldosterone—and the changes in adrenal miRNA expression that accompany this disruption. The main evidence of this nature comes from our own group, initially using the H295R human adrenocortical cell line. Using siRNA, we knocked down H295R expression of Dicer1, a key RNAseIII enzyme that generates miRNAs from their pre-miRNA precursors. This general reduction in miRNA levels effectively de-repressed cellular levels of specific messenger RNAs encoding key enzymes required for aldosterone biosynthesis, including *CYP11B2* (aldosterone synthase) [18]. Furthermore, Dicer1 knockdown also resulted in a 50% increase in cellular aldosterone levels, implying that adrenal miRNAs have a general inhibitory effect on aldosterone biosynthesis. More detailed analysis established that a specific miRNA, hsa-miR-24-5p (technically two miRNAs of identical sequence, hsa-miR-24-1-5p and hsa-miR-24-2-5p, transcribed from two different sites), was capable of binding *CYP11B2* mRNA, thereby repressing its expression; subsequently, we found miR-125a-5p and miR-125b-5p also repressed *CYP11B2* [19]. In our hands, we found no evidence that hsa-miR-10b-5p directly regulates *CYP11B2* [18], although Nusrin et al. reported contradictory findings using the same cell model, also showing expression of this miRNA to be induced under hypoxic conditions [20]. More recently, Zhang et al. also reported direct targeting of *CYP11B2* by hsa-mir-193-3p [21]. However, the influence of miRNA on the aldosterone steroidogenic pathway is not confined to *CYP11B2*; we have also shown significant effects on *CYP11A1* and *CYP21A2* [19], and it would seem likely that miRNAs are able to control this specific pathway at various stages, even before one considers their influence over the various regulatory mechanisms (primarily the renin–angiotensin system) that stimulate the adrenal cortex to produce aldosterone. Therefore, it seems likely that we have only scratched the surface of the numerous ways in which miRNAs intervene in the regulation, synthesis and action of aldosterone, either in the adrenal cortex or elsewhere [22,23,24].

In order to demonstrate that PA changes the levels of specific miRNAs in the adrenal glands of affected patients, we also undertook miRNA profiling that compared APA with nontumorous adrenal tissue. Due to the technologies available at the time, this study was small (*n* = 4 for each group) and current analytical methods are superior to the microarrays that were used (see below). Nevertheless, this study did show levels of many miRNAs to be highly correlated between the two tissue types, with several having significantly divergent expression, including the aforementioned hsa-mir-24-5p and hsa-mir-10b-5p, both of which were downregulated in APA relative to normal tissue [18]. A separate study by He et al. identified significant differential expression of 31 miRNAs in APA or unilateral adrenal hyperplasia tissue relative to normal adrenal cortex using microarray technology [25]. They confirmed downregulation of hsa-miR-375 by qRT-PCR and presented evidence that this miRNA may act as a suppressor of tumour growth through its repression of *MTDH* expression. Velázquez-Fernández et al. sought to distinguish subtypes of adrenocortical adenoma (ACA) on the basis of their tissue miRNA profiles. They detected and quantified miRNA in APA (*n* = 9), cortisol-producing adenoma (CPA, *n* = 10) and non-hyperfunctioning adenoma (NHFA) samples by microarray and were able to group these three subtypes according to their miRNA profiles [26]. Each subtype was also compared to “normal” adrenal reference samples and, relative to these, APAs were found to have eight miRNAs that were significantly over-expressed and four that were under-expressed in this subtype alone (although these miRNAs did not include hsa-mir-24-5p, hsa-mir-10b-5p or hsa-miR-375). More specifically, Lenzini et al. identified APA-expressed miRNAs likely to target directly the expression of TASK-2, a K^+^ channel whose downregulation is observed in APA and which apparently drives its pathogenic effects [27]. They found hsa-miR-23 and hsa-miR-34a to fulfil this role, modulating TASK-2 levels in APA in a manner likely to enhance aldosterone secretion through increased expression of *CYP11B2* and the steroidogenic acute regulatory protein (*STAR*). Peng et al. showed hsa-mir-203 to be downregulated in APA relative to adjacent adrenal tissue and confirmed that this miRNA targets *WNT5A*, a component of the Wnt/β-catenin pathway; by manipulating levels of miR-203 within APA cells, they could alter aldosterone production and cell proliferation [28]. These different studies not only provide insight into the miRNA-mediated mechanisms underlying aldosterone secretion, but also establish the possibility of identifying and distinguishing the tumorous APA tissue from normal adrenal tissue by their miRNA profiles due to the disruption of these same mechanisms.

Such changes between APA and non-diseased adrenocortical tissue, together with the knowledge that miRNAs participate in the control of aldosterone biosynthesis, are promising indicators that c-miRNA biomarkers for PA might exist. The simplest hypothesis would be that such adrenal changes in miRNA levels are reflected directly in the circulation, with levels of specific miRNAs rising or falling as they do in the adrenal cortex. However, it may not be a straightforward question of simply measuring these miRNAs in the bloodstream. For example, the quantities of a potential biomarker miRNA released from the adrenal gland may not be sufficient for it (or any changes in its quantity) to be measured reliably in the circulation. Alternatively, circulating quantities of that miRNA may be supplemented—or completely swamped—by release of the same miRNA from other tissues, preventing any detection of an adrenal-specific signal. Additionally, a viable c-miRNA biomarker for PA may not necessarily derive from the adrenal gland at all. Rather, PA could trigger miRNA changes in other affected tissues, such as the vasculature, which might be better candidate biomarkers than adrenal miRNAs. For these reasons, although studies of adrenal tissue are encouraging, identification of potential diagnostic miRNA biomarkers requires careful direct analysis of the miRNAs circulating in the bloodstreams of PA patients.

## 4. Circulating miRNA Studies of Hypertension and PA

A high-throughput biomarker-based diagnostic test that distinguishes PA patients from those with other forms of hypertension is clearly desirable. At present, we are unaware of any studies that have directly compared c-miRNA profiles of PA patients with those of essential hypertensives. Nevertheless, certain previous c-miRNA studies do provide useful insights.

The only major study to date examining c-miRNA profiles in the context of PA was published by Decmann et al. in 2019. Rather than attempting to diagnose PA as a single entity, they instead sought to distinguish the two principal forms of PA—BAH and APA—from one another on the basis of c-miRNA [29]. To this end, RNA-seq was first conducted on 30 plasma samples (16 APA and 14 BAH), identifying an initial list of 50 c-miRNAs that were significantly differentially expressed between groups. Attempted validation of the four most statistically significant of these by qRT-PCR in a further 93 samples confirmed differential expression of three c-miRNAs—hsa-miR-30e-5p, hsa-miR-30d-5p and hsa-miR-7-5p—all of which were upregulated in BAH relative to APA. Diagnostic performance of the three c-miRNAs, as assessed by ROC analysis, showed the specificities and sensitivities of each to be approximately 60% at the chosen (presumably optimal) cutoff points. While the authors correctly noted that this is poor in comparison to AVS (with sensitivity of 92.5% and specificity of 100%), the study findings are important in several respects. Firstly, the relatively large number of well-phenotyped samples analysed using RNA-seq provides a comprehensive survey of the c-miRNAs present in PA patients. Secondly, the great care taken in design and conduct of the study meant that, of just four miRNAs tested by qRT-PCR, three were successfully validated; there is therefore a strong possibility that levels of several more c-miRNAs are significantly altered between APA and BAH subjects. Additionally, as the diagnostic utility of the three c-miRNAs was only assessed individually, it may be that combination of these miRNAs—plus other differentially expressed miRNAs yet to be validated—into a “signature” could significantly improve the performance of the test. Finally, as the investigators point out, adrenal glands in the bilateral form of disease are now known to harbour cell lesions with mutations that drive aldosterone production [3], blurring the boundary between BAH and APA. If the pathologies of these PA subtypes are really so closely related, then it is impressive that any consistent and significant difference was apparent at all and implies that the ostensibly simpler task of distinguishing PA (regardless of subtype) from essential hypertension might yield results of greater clinical relevance. Unfortunately, given that the study population was composed entirely of PA subjects, the findings of this study provide no indication of which individual c-miRNAs may be of diagnostic value in distinguishing PA from essential hypertension. It is also interesting to note that, for reasons unknown, patient samples contributed by different study centres were shown to differ significantly in their miRNA levels, as measured by qRT-PCR, underlining the need to standardise sample collection and storage in order to avoid bias even before sample processing.

Another recent study compared c-miRNA in the plasma of essential hypertensives and healthy (i.e., normotensive) individuals drawn from the Uyghur population of Northwest China. Initially, microarray was used to analyse a small number of samples (*n* = 4/group), identifying 257 differentially expressed c-miRNAs [30]. Of these, 161 were upregulated in hypertension, while 96 were downregulated; the upregulated miRNAs showed a much greater degree of fold change. Although it is tempting to conclude from this that the dysfunction associated with hypertension leads to large and readily detectable dynamic changes in levels of certain c-miRNAs, subsequent qRT-PCR validation found the fold changes to be far smaller and suggests such large observed shifts may be an artefact of microarray quantification. Just 4 of the most significant miRNAs were selected for qRT-PCR validation (*n* = 15/group), but all were confirmed, with hypertensives having significant upregulation of hsa-miR-198 and hsa-miR-1183, and downregulation of hsa-miR-144-3p and hsa-miR-30e-5p (note that this last c-miRNA was found to upregulated by Decmann et al. in BAH vs. APA [29]). The microarray data were used as the basis of Hierarchical Clustering analysis, which was able to group samples clearly into hypertensive and normotensive classes on the basis of increased and decreased levels of specific miRNAs. Clearly, it would be interesting to confirm whether this relationship is maintained using a larger study sample, more reliable quantitative data and other ethnic groups. It is to be hoped that improvements in methodology and greater standardisation of workflows will permit such replication but data from previous studies of c-miRNA in essential hypertension are not promising, with previous overviews of this topic highlighting the inconsistent study results [31].

In summary, while previous studies of PA and essential hypertension suggest disruption of the c-miRNA profile in a manner that might be exploited for diagnostic purposes, it is apparent that inconsistent methodologies present a significant obstacle, obscuring our ability to discern the true and specific effects, if any, of PA on the circulating miRnome.

## 5. Technical Challenges in the Design of Circulating miRNA Studies

Throughout the last decade, numerous studies have attempted to establish diagnostic c-miRNA profiles for a diverse range of conditions. Yet, even studies that analyse identical conditions have struggled to reach significant agreement in their findings. Such lack of reproducibility appears to be due mainly to differing methodological approaches [32]. This leads to variation in miRNA quantification and, ultimately, inconsistency in those individual miRNAs found to associate significantly with a particular disease. Therefore, any studies attempting to define diagnostic c-miRNA signatures, whether it be for PA or other conditions, must be designed in order to remove, or at least minimise, the impact of these confounding factors, which can affect every stage of the process. Additionally, any study that seeks to confirm or verify the findings of earlier work should seek to recreate that methodology as closely as is practical. Before discussing some of the existing c-miRNA studies relevant to PA, it is necessary to describe these confounding factors and how they influence good study design (see summary of factors in Table 1).

### 5.1. Study Population

As with any clinical study, it is important to ensure that patient and control populations are carefully phenotyped in order to ensure their correct classification. In the case of a study seeking to distinguish PA patients from essential hypertensives, this is particularly difficult as, based on the emerging evidence of PA underdiagnosis, it is likely that any group of essential hypertensives will in fact harbour a significant proportion of undiagnosed PA subjects, which might frustrate attempts to clearly distinguish them from the diagnosed PA group on the basis of c-miRNA profile. Levels of c-miRNAs are known to associate with such characteristics as sex (e.g., hsa-miR-150-5p and hsa-miR-145-5p), age (e.g., hsa-miR-126-3p and hsa-miR-21-5p) and BMI (e.g., hsa-miR-122-5p, hsa-miR-148-3p and hsa-miR-505-3p), so any study populations must also be carefully matched for these if misleading conclusions are not to be drawn [33].

### 5.2. Sample Type

The analysis of cell-free miRNA (cfmiRNA) in the circulation requires the investigator to decide between plasma or serum as the sample medium of choice. Alternatively, investigators may choose instead to investigate c-miRNAs that have been secreted into extracellular vesicles (EVmiRNA). While it may appear obvious that cfmiRNA and EVmiRNA studies will significantly detect different arrays of miRNAs, reflecting their very different sources [34], it should be noted that plasma and serum might also yield broadly similar but different results despite both capturing the cfmiRNA fraction [35]. This underlines the fact that meaningful comparisons cannot necessarily be made between otherwise highly similar cfmiRNA studies that employ different sample media. There is also some evidence that the process of coagulation required for the preparation of serum leads to contamination of the sample with haemolysis-derived miRNAs, implying plasma may be the superior biofluid [35]. Comparisons between EVmiRNA studies are even more problematic, as different protocols will isolate EVs of differing properties (e.g., size, density, solubility) and, consequently, different miRNA composition [36].

### 5.3. Sample Storage and Quality

High-quality cfmiRNA samples should suffer minimal degradation prior to analysis and should also not be contaminated with cellular miRNAs that might skew results. Therefore, plasma or serum samples should not be significantly haemolysed and might be excluded on the basis of visual/spectrophotometric inspection or high levels of miRNAs thought to be of erythrocyte or platelet origin [37]. Heparin should be avoided as it has been shown to interfere with downstream miRNA detection [38]. It is desirable to isolate miRNA from blood samples immediately upon collection, although this may not always be possible in a clinical setting. Instead, plasma/serum should be prepared from whole blood as soon as possible after sampling (<2 h) in order to minimise haemolysis, before freezing, preferably at −80 °C. MiRNA stability under such storage conditions is very good—and superior to that in whole blood—but multiple freeze/thaw cycles should be avoided [39]. Post-isolation from plasma or serum, miRNA itself appears highly stable for many years at ultra-low temperature and through multiple freeze/thaw cycles [40].

### 5.4. miRNA Isolation Method

A diverse variety of methods are available for the isolation of miRNA from plasma/serum, varying in such key factors as their use of filter columns and/or phenol for extraction. Numerous studies have demonstrated the significant impact of different RNA isolation protocols on the quantified levels of serum/plasma miRNAs, emphasising that consistent methodology must be employed throughout individual studies [41]. In addition to protocol differences, operator variability may also be a factor. For example, Kloten et al. compared the performance of seven different protocols for the extraction of cfmiRNA and EVmiRNA from plasma. Analysis of the resulting RNA samples by real-time qRT-PCR and NGS showed clear differences in the measured quantities of specific miRNAs, which were attributed mainly to the variable efficiency of each miRNA extraction method. However, given that each method was performed at one of six different centres, operator variability was possibly also a factor [42].

### 5.5. Quantification Method

The choice of method employed to quantify miRNAs extracted from liquid biopsy samples is determined to a large degree by the range of miRNAs one wishes to detect, the accuracy of quantification one wants to achieve and overall cost. The pros and cons of different methods have been well reviewed elsewhere [41] but it is worth summarising the main options here in order to draw attention to the challenges of accurately quantifying the diverse range of c-miRNA species accurately. Added to this are the different requirements of initial screening studies that aim to survey as much of the c-miRNA transcriptome as possible versus those of a final diagnostic test, designed to quantify only a select number of miRNA species accurately and reproducibly.

Initial studies of c-miRNA species tended to use microarray technology, which enabled detection of several hundred miRNAs simultaneously in a single sample. The main drawback to this method is its semi-quantitative nature: while useful for initial identification of shifts in miRNA levels, any such changes required validation using a fully quantitative method such as realtime quantitative RT-PCR (RT-qPCR). Given that such validation often fails to confirm the microarray results, presentation of microarray data without this supporting evidence should be treated with caution. This confirms the status of RT-qPCR as the gold standard for quantitative measurement of miRNA, although the protocol for small RNAs is not so straightforward as for mRNA. MiRNAs must be enzymatically treated prior to reverse transcription (RT) in order to attach additional linker sequences that provide sufficient length for the annealing of PCR primers. This can introduce bias into the RT stage due to the variable efficiency of linker attachment. PCR assays themselves are generally sensitive and specific, although there is a risk of false positive results where a given assay fails to distinguish two highly similar miRNA species. Specificity can be improved through the use of locked nucleic acid (LNA) primers, which have greater binding stability than DNA, enabling the use of shorter sequences. The greatest drawback of RT-qPCR is the need to perform a separate assay for each individual miRNA species which, at least in the early discovery stages of a study, may be highly intensive in terms of operator time, cost and template RNA. The inconvenience of this can be reduced by the use of array plates (e.g., QIAGEN LNA miRNA Focus PCR panels), which assemble numerous different qPCR assays on to a 384-well plate format. On the other hand, a distinctive miRNA diagnostic “signature” consisting of, for example, five or six different miRNAs, would not be such an issue, and qRT-PCR would be by far the most practical, efficient and reliable method for such assays.

Finally, there is RNA-Seq which, due to significant reduction in the costs of next-generation (NGS) sequencing technology over the last decade, has become a much more viable tool for discovery studies that wish to survey the whole of the c-miRNA transcriptome, though still expensive and requiring a great deal of downstream data analysis. The major advantage of RNA-Seq is its ability to detect the presence of novel or unexpected miRNAs in a sample, as opposed to microarray and RT-qPCR where measurement is confined to known miRNA species selected in advance (and to the capacity of the plate/chip, although several hundred miRNAs can potentially be assayed in a single run). NGS requires the assembly of a library from the original RNA sample, and this can be the source of considerable bias and variability, as can batch effects where technical variability between machine runs makes comparison of samples across larger studies challenging. Additionally, as for microarray, RT-qPCR validation of any quantitative shifts is necessary. However, this area of technology is developing rapidly, and RNA-Seq is likely to be the main tool, at least for large-scale analysis of diverse miRNAs, in the future. Relative to the other technologies, it should be borne in mind that RNA-seq produces large datasets requiring a great deal of operator and computer time to process, using workflows that are not yet fully standardised in order to map sequencing reads to genomic databases of miRNA sequences [41]. This, again, is an important practical consideration to take into account when designing c-miRNA studies.

### 5.6. Quality Control/Normalisation of Data

Variation in the quality and quantity of starting RNA and also in the efficiencies of the various stages of the quantitation process—RNA isolation, RT, PCR—mean that data derived from multiple samples must be normalised to a common reference. However, at present, there is no standard normalisation procedure for c-miRNA and such data correction must be applied carefully if it is not to produce misleading results [43]. Exogenous reference miRNAs (also known as spike-in controls) can be introduced in known quantities to the sample at various stages. In human studies, these tend to be synthetic sequences that mimic miRNAs from other species such as *C. elegans* that have no human counterpart, in order to avoid confusion with endogenous sequences. An alternative approach has been to separately amplify known concentrations of exogenous miRNAs, in synthetic form, alongside the study samples. Standard curves can then be constructed that enable quantification of these specific miRNAs in the samples [44]. These are highly useful ways of confirming the efficiencies of the various technical stages, thereby maintaining quality control and ensuring the consistency of these processes across an entire study. However, they do not permit normalisation for variation in the quality and quantity of the original RNA sample, for which an endogenous miRNA (or miRNAs) is required to act as reference. Unfortunately, there is no consensus as to which c-miRNAs, if any, are sufficiently stable to serve as a reference in all situations, enabling normalisation of data on the basis of minor shifts in RNA quality/quantity, and also flagging outlying samples containing RNA that is degraded or at a concentration outside acceptable limits. At present, it is therefore necessary to quantify a number of endogenous miRNAs across all samples and then identify those which show the most stable expression on the basis of the delta Ct method or using specialist software such as NormFinder, geNorm or BestKeeper. These packages also permit multiple miRNAs to be used as normalisers, reducing error due to small fluctuations in a single reference and enhancing the robustness of data normalisation. Although this approach has identified certain c-miRNAs that are stably expressed in biofluids in multiple studies (e.g., hsa-miR-16-5p, hsa-miR-93-5p, hsa-miR-191-5p), it is doubtful that any one of these could be used universally [45]. So, at least for the immediate future, normalising c-miRNAs must be identified on a study-by-study basis.

### 5.7. Data Analysis

Standard statistical analysis can be used to highlight those c-miRNAs present at readily detectable levels in patient and/or control samples as well as identifying those that are significantly differentially expressed between the two. While this is a valid approach to identifying candidate diagnostic c-miRNAs on an individual basis, it is suboptimal when trying to establish the optimal combination of biomarker c-miRNAs into a diagnostic “signature” of high sensitivity and specificity. Such analysis requires the use of multivariate methods. c-miRNA studies can benefit from the lead taken by proteomic biomarker studies in this regard, where a variety of both unsupervised pattern recognition methods (e.g., principal component analysis, hierarchical clustering) and unsupervised methods (e.g., Bayesian methods and machine learning) have been employed [46]. Clearly, such work requires close collaboration with specialised statistical experts.

## 6. Study Design and the ENS@T-HT Project

The previous section serves to emphasise the numerous choices faced in the development of c-miRNA studies and while many of these options may not be objectively superior to their alternative, it is apparent that they could have significant impact on the study findings. Therefore, we should perhaps not be surprised that there has been such apparent lack of correlation even between superficially similar c-miRNA studies given that their methodologies are likely to have diverged in significant ways. Evidently, to determine whether a c-miRNA-based diagnostic test for PA is viable, we require a comprehensive study to be conducted utilising, as far as is practicable, the best and most consistent methods for quantifying and analysing c-miRNAs so as to develop an identifying signature. This signature can then be tested in an independent population using the same methodology as confirmation of consistent and reproducible shifts in c-miRNA levels.

**Figure 1:**
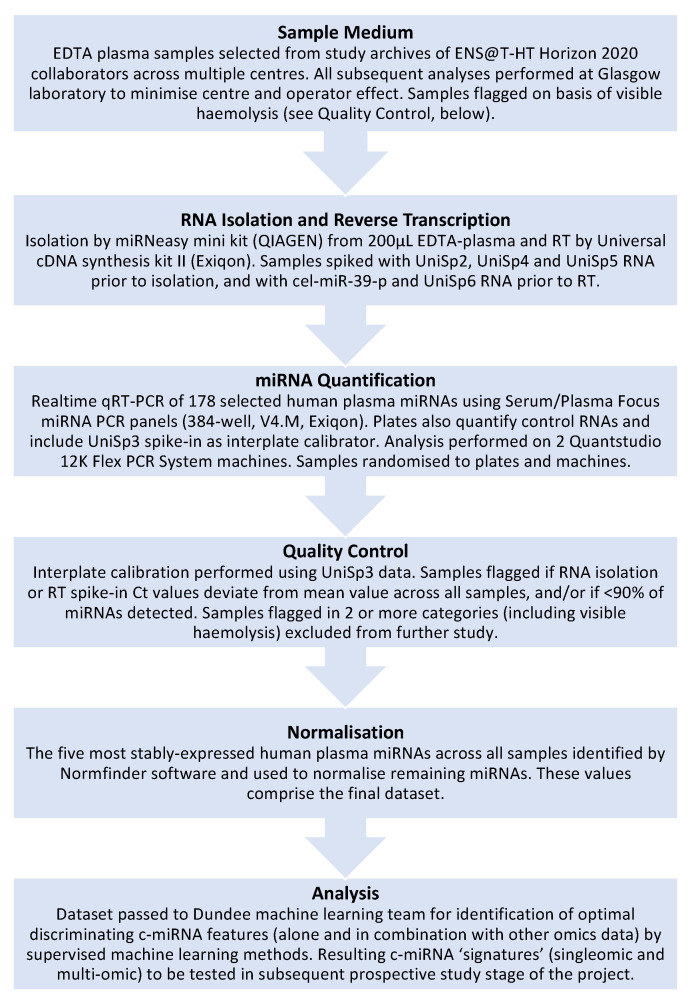
Summary of workflow for c-miRNA analysis in the retrospective ENSAT-HT study.

This is the thinking behind ENS@T-HT, an EU-funded Horizon 2020 research and innovation project designed to develop diagnostic signatures for various forms of endocrine hypertension—including PA, Cushing’s syndrome (CS) and phaeochromocytoma/functional paraganglioma (PPGL)—on the basis of patient profiles that have been defined using various “-omics” approaches (www.ensat-ht.eu, accessed on 30 August 2021). These omics include measurements of steroids, small metabolites and metanephrines in plasma, as well as urinary steroids, and—most relevant here—c-miRNAs. The ultimate aim is to combine the most informative measurements from these various omics into an optimal multiomic signature that would form the basis of a single high-throughput diagnostic test that could be performed readily at non-specialist clinical centres. The study consists of two distinct stages—the retrospective phase and the prospective phase—each of which involves the analysis of a completely separate and independent study population; as these names imply, the samples analysed during the retrospective phase were collected over a period of years prior to the study (*n* = 357, plus normotensive volunteers as comparators), while the prospective phase analyses samples collected specifically for ENS@T-HT (*n* > 1000). The intention is first to define a diagnostic signature in the retrospective population that can then be validated in the prospectively collected subjects. Our group has led the c-miRNA arm of this project and has therefore been required to make several key decisions regarding the c-miRNA quantification/analysis pipeline that must balance several occasionally conflicting considerations. Furthermore, during our design of the retrospective study, we were mindful of the ultimate purpose of the project, which is to develop a diagnostic assay that can be rapidly and reproducibly employed in non-specialist healthcare centres using commonly available laboratory equipment. These considerations are summarised below and in Figure 1.

### 6.1. Study Subjects

For the retrospective study, samples from >350 patients (male or female, aged 11–78y) have been studied, each allocated to one of the four groups: PA, CS, PPGL and primary hypertension (PHT). Although secondary hypertension had been ruled out as a factor in some of the PHT subjects within the retrospective phase, the possibility does remain that a minority of the PHT group may include either “pre-PA” or undiagnosed PA subjects. This possible confounder in the identification of diagnostic biomarkers for PA is unfortunate but largely unavoidable owing to the shortcomings in PA diagnosis that we aim to address. We depend upon our study size and analytical methods to reduce the impact of this effect.

### 6.2. Sample Medium

EDTA-plasma has been used as the biofluid of choice throughout this project, as it was judged that this would be the simplest medium to collect, store and process in a “real-world” clinical environment (as opposed to the more complex procedures for serum and microvesicles) and would, therefore, reduce inter-sample variability.

### 6.3. miRNA Quantification Method

The use of RNAseq for this large number of subjects was deemed impractical due to practical considerations (e.g., the considerable amount of data generated) and the prohibitive cost. For this reason, we instead settled on using a realtime qRT-PCR array plate system (Exiqon Serum/Plasma Focus microRNA PCR Panels), which would offer robust quantitative data across 178 endogenous circulating miRNAs, as well as the necessary exogenous control miRNAs. While this approach does not permit the identification of novel c-miRNAs, it does remove the need for subsequent validation of quantitative data, as would be required for RNAseq or microarray analysis. Additionally, quantification can be performed on standard realtime thermal cyclers (we used the QuantStudio 12K Flex Real-Time PCR System, Thermo-Fisher, Waltham, MA, USA), which is common laboratory equipment likely to be available in the diagnostic laboratories that are the intended end-users of this test. Therefore, this approach provides further insight into whether measurement on this type of equipment is sufficiently sensitive to be practical in a clinical setting.

### 6.4. RNA Isolation Method/RT/Quality Control/Normalisation Methods

The choice of quantification method dictated to some degree the RNA isolation and RT methodologies used (QIAGEN miRNeasy mini kit and Exiqon Universal cDNA synthesis kit II, respectively), as it was necessary to ensure all were compatible and provided sufficient yield, purity, etc., for quantification. Total RNA was isolated from 200 microlitres of total plasma in all cases and subsequent volumes carried over for RNA elution, cDNA synthesis, etc., were standardised throughout the process to minimise as far as possible any inter-sample variation. In addition, plasma samples were seeded with three different spike-in miRNAs (UniSp2, UniSp4 and UniSp5), and an additional two miRNAs (cel-miR-39-3p and UniSp6) were added to RNA prior to cDNA synthesis, thereby providing measures for efficiency of RNA isolation and reverse transcription. The order in which samples were run on the QuantStudio equipment was randomised in order to reduce possible batch effects that might emerge over the course of the project. Prior to normalisation, outlier samples were excluded from further analysis; these included samples where levels of spike-in control miRNAs deviated significantly from the mean and samples where >10% of endogenous c-miRNAs were not detected. In addition, c-miRNAs that were not detected in >50% of all samples were not subjected to further analysis. Remaining miRNA data from samples that had passed these quality control thresholds were subjected to normalisation using Normfinder, which is proven to offer robust correction of minor variation in such quantitative measurements [47,48]. As mentioned previously, Normfinder is a software package that identifies the most stably expressed c-miRNAs across all analysed samples and uses these to normalise the levels of the remaining endogenous miRNAs. Using this software, we have used a combination of five stably expressed c-miRNAs to normalise the quantification data.

### 6.5. Analytical Methods

Given the complex multivariate analyses required to evaluate the numerous molecules measured by each omic approach within the ENS@T-HT project (as well as the combination of each omic into a single signature), a machine learning approach has been implemented to define each endocrine hypertension disease signature relative to an essential hypertensive group. While it is anticipated that this multiomic approach will yield the most informative signature for each condition, individual omics (i.e., singleomics, including c-miRNA) are also being analysed separately to assess their diagnostic utility. The role that significant factors might play in disease aetiology will also be investigated.

To date, miRNA measurement and analysis has been completed for the retrospective population, providing a reduced list of potential biomarker c-miRNAs that have now also been quantified in the prospective population using the same methodologies as for the retrospective phase. Analysis of these data by machine learning methods is ongoing. Despite spending several years in freezer storage, the quality of miRNA isolated from retrospective study samples was generally high and not appreciably different from that of the prospective samples, which had been frozen for only a few months, at most.

The above is intended to give some insight into the potential confounders of consistent measurement that we have sought to address and minimise during a large project designed to discover c-miRNA biomarkers. It is obviously our hope that there will be significant correlation between the findings of the retrospective and prospective studies, providing reproducible evidence for the first time that c-miRNAs can serve as diagnostic molecules for various forms of endocrine hypertension, including PA, either as a singleomic signature or, more likely, as components of a multiomic signature that also encompasses other types of molecules, including steroids, small metabolites and metanephrines.

## 7. Conclusions

Improved diagnosis of PA would yield significant benefits, reducing cardiovascular morbidity and mortality through the early diagnosis and treatment of the many hypertensives whose underlying pathology is presently unidentified. Here, we have described the role that miRNAs play in regulating steroidogenic processes within the adrenal cortex and the disruption to its miRNA transcriptome that occurs in PA. This lends compelling support to the hypothesis that such changes in adrenal tissue (and elsewhere) may be reflected in the array of c-miRNAs in the bloodstream. Molecular laboratory methods have now reached the point where such c-miRNAs can be routinely and accurately quantified using commonly available laboratory equipment. The development of a rapid diagnostic test for PA utilising c-miRNAs that could be performed routinely in non-specialist centres is, therefore, a highly desirable and potentially achievable goal. However, as is apparent from previous studies of c-miRNAs in the context of numerous conditions over the past decade or so, there is still some doubt as to whether circulating levels of miRNAs linked to particular diseases show sufficient specificity and sensitivity to be employed as effective biomarkers. It is the belief of ourselves and others that a great deal of the observed variability and lack of reproducibility are the result of inconsistent and non-standardised practices at various stages of the c-miRNA quantification procedure. Therefore, we argue that the validity of c-miRNAs as PA biomarkers should be tested in two independent populations using identical methodology. We are currently engaged in conducting such a study under the umbrella of the ENS@T-HT project. The design of this study has been carefully considered in order to minimise confounding variability in c-miRNA quantification, while also utilising approaches that can be easily performed in a non-specialist clinical setting. The full data from this study are now undergoing final analysis. It is our hope that its findings will establish a firm evidence base for the use of c-miRNAs as diagnostic biomarkers for PA.

## Figures and Tables

**Table 1:** Factors to be considered in the design of circulating miRNA analysis study protocols. These have the potential to affect outcome by influencing the number and identities of differentially expressed miRNAs detected.

Study Stage	Factors
Study population	Careful phenotyping and correct allocation to study groups Matching of groups for potentially confounding characteristics, e.g., sex, age, BMI, etc.
Sample type	Choice of cell-free or extracellular vesicle-bound miRNA Choice of biofluid, e.g., plasma or serum?
Sample storage	Minimal haemolysis of serum/plasma samples Avoidance of prolonged sample storage at room temperature Avoidance of freeze/thaw cycles
miRNA isolation method	Choice of miRNA isolation protocol/kit Consistent protocol to be maintained throughout study and across all centres Potential for operator effect?
Quantification method	Choice of methodology dictated by various factors including sample number, throughput, cost, accuracy/sensitivity, data analysis support Available methods include RNA-Seq, realtime qRT-PCR (incl. array plates), microarray.
Quality control/data normalisation	Identification of poor quality/degraded samples Normalisation of data to account for variation across different centres, machine runs, operators, etc. Use of spike-in miRNAs at various points during sample analysis protocol Identification of stably expressed endogenous miRNAs that can be employed as normalisers (singly or in combination)
Data analysis	Standard statistical methodology to identify differential expression Establishment of optimal predictive/diagnostic miRNA combinations (‘signatures’) through multivariate analysis methods
